# HMGB1 in Systemic Lupus Erythematosus

**DOI:** 10.3389/fimmu.2020.01057

**Published:** 2020-05-27

**Authors:** Tianye Liu, Myoungsun Son, Betty Diamond

**Affiliations:** ^1^Center for Autoimmune Musculoskeletal and Hematopoietic Diseases, Institute of Molecular Medicine, The Feinstein Institutes for Medical Research, Manhasset, NY, United States; ^2^Donald and Barbara Zucker School of Medicine at Hofstra/Northwell, Hempstead, NY, United States

**Keywords:** HMGB1, SLE, neuropsychiatric SLE, lupus nephritis, innate immunity, adaptive immunity

## Abstract

The high-mobility group box 1 (HMGB1) has been shown to exert proinflammatory effects on many cells of the innate immune system. Originally identified as a nuclear protein, HMGB1 has been found to play an important role in mediating inflammation when released from apoptotic or necrotic cells as a damage-associated molecular pattern (DAMP). Systemic lupus erythematosus (SLE) is a disease of non-resolving inflammation, characterized by the presence of autoantibodies and systemic inflammation involving multiple organ systems. SLE patients have impaired clearance of apoptotic debris, which releases HMGB1 and other DAMPs extracellularly. HMGB1 activity is implicated in multiple disease phenotypes in SLE, including lupus nephritis and neuropsychiatric lupus. Elucidating the various properties of HMGB1 in SLE provides a better understanding of the disease and opens up new opportunities for designing potential therapeutics.

## Introduction

### Systemic Lupus Erythematosus (SLE)

SLE is an autoimmune disease characterized by the production of autoantibodies and multi-organ system involvement with a wide array of clinical manifestations. The dominant clinical features include fever, arthritis, serositis, cutaneous lesions, neuropsychiatric and renal involvements (1). SLE is caused by aberrant activation of autoreactive B cells and subsequent production of autoantibodies against nucleic acid and nucleic acid binding proteins. These bind to tissue, often through cross-reactivity to a tissue antigen, and cause organ damage (2). Immune complexes containing nucleic acid can be internalized through Fc receptor engagement and activate cells of the innate immune system. Thus, neutrophils, monocytes, macrophages, and dendritic cells (DCs) contribute to SLE pathologies (3), in part following, cytosolic sensing of DNA or RNA in part through an impairment in the usual non-immunogenic clearance of apoptotic debris, and in part due to cell intrinsic genetic alterations.

Genetic factors in the context of environmental triggers are thought to play important roles (4). Some identified risk gene loci for SLE include BLIMP1, IRF5 and C1q (5–8). C1q binds to opsonized cellular debris to mediate the clearance of dead and dying cells (9–11). Genetic deficiency of C1q predisposes strongly to SLE (7, 12, 13). SLE occurs in approximately 90% of C1q-deficient individuals in many studies. These patients have severe central nervous system and renal autoimmune disease.

### High-Mobility Group Box 1 Protein (HMGB1) in SLE

**Figure 1:**
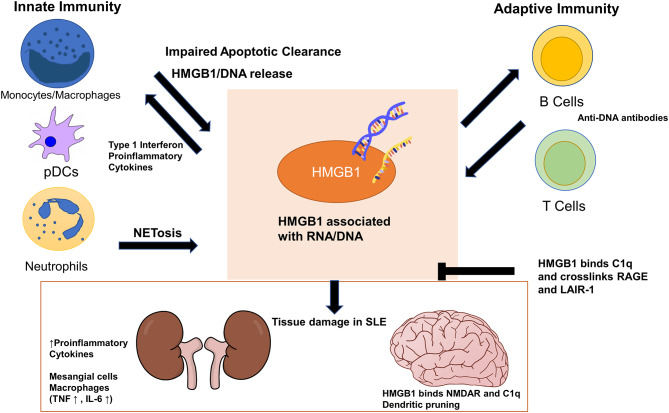
High-mobility group box 1 (HMGB1) exerts its pathogenic effects in systemic lupus erythematosus (SLE) through cells in both the innate and adaptive immune systems. Impaired apoptotic clearance by macrophages prolongs exposure of HMGB1/nucleic acid-containing debris to the adaptive immune system as autoantigens. HMGB1 enhances adaptive immune response in generating autoantibodies against DNA/RNA/HMGB1, which cross react with tissues and cause organ damage. HMGB1 also locally increases proinflammatory cytokines and stimulate mesangial cells and macrophages in the kidneys. In the brain, HMGB1 bridges binding of C1q to N-methyl-D-aspartate receptor (NMDAR) to promote dendritic pruning and spatial memory deficit. HMGB1 can stimulate monocytes and plasmacytoid dendritic cells to sustain the production of type 1 interferon seen in SLE. HMGB1 can perpetuate its extracellular presence both by inducing more HMGB1/DNA release from neutrophil NETosis and by deviating macrophage polarization away from M2, which further impairs apoptotic clearance. HMGB1 and C1q together crosslink RAGE and LAIR-1 to exert anti-inflammatory and pro-resolving effects on monocytes.

HMGB1 is a member of the family of high-mobility group (HMG) proteins which were identified as important non-histone nuclear proteins (14, 15). Also known as amphoterin, HMGB1 has a molecular weight of 25 kDa and two positively charged nucleic acid binding motifs, A box and B box, and a negatively charged C-terminal tail (16, 17). The function of HMGB1 in the cell is context-dependent. In the nucleus, HMGB1 plays the essential role of bending DNA and facilitating its interaction with transcription factors. HMGB1 can also function as a DAMP outside the cell, activating the immune system and promoting inflammation. It is released from damaged cells or activated cells to exert its inflammatory effects (18). It binds both receptor for advanced glycation endproducts (RAGE), toll-like receptor 2 (TLR2) and TLR4 (19–21). There are three cysteine residues (C23, C45, C106) in HMGB1 and their redox states dictate the function of HMGB1. Histone H1 is most effective at inhibiting the DNA bending activities of oxidized HMGB1 (22). Additionally, HMGB1 oxidation is known to alter its extracellular receptor binding and subsequent functions. As reviewed by Janko et al., fully reduced HMGB1 can induce autophagy through binding to RAGE or together with CXCL12 can promote cell migration through binding to CXCR4. When C23 and C45 are oxidized to form a disulfide bond, HMGB1 can signal through TLR4 and cause pro-inflammatory cytokine release (23). Oxidative stress is known to be increased in SLE and contributes to immune system dysregulation (24) and it is likely that partially oxidized, disulfide HMGB1 contributes to this process. Serum HMGB1 is elevated in SLE patients and levels of serum HMGB1 correlate with disease activity (25). The present review discusses the role of HMGB1 as a DAMP in both the innate and the adaptive aspects of SLE pathogenesis (Figure 1).

## HMGB1's Role in SLE Pathogenesis

### Adaptive Immunity

Antibodies to nuclear antigens are the hallmark of SLE (26). These autoantibodies to ubiquitous self-antigens lead to immune complex formation, to deposition in tissue and ensuing tissue damage. Apoptotic defects are an important aspect of SLE pathogenesis (27). When apoptotic cells are not efficiently cleared, they can undergo secondary necrosis, releasing their intracellular contents (28). The HMGB1 released in this process can play a role as an autoadjuvant in the breakdown of B cell tolerance and the generation of autoantibodies in SLE. As HMGB1 can bind both RNA and DNA it can activate cytosolic nucleic acid receptors after entering cells in a RAGE dependent fashion (29). It has been shown *in vivo* that HMGB1-nucleosome complexes activate antigen presenting cells and elicit an anti-dsDNA and anti-histone IgG response in a TLR2-dependent manner, whereas HMGB1-free nucleosome do not (30). Although anti-nuclear antibodies (ANA) in SLE most commonly bind to DNA and histones in nucleosomes, they are also reported to bind to HMGB1 itself (31, 32), although this may represent binding to DNA associated with HMGB1. Elevated anti-HMGB1 antibodies are observed in SLE and correlate to disease severity (33, 34). Coupled with elevated circulating HMGB1 seen in SLE patients, this can be a mechanism for immune complex formation that includes nucleic acid which is bound to the HMGB1.

### Innate Immunity

Although the adaptive immune system has been studied extensively for its roles in producing autoreactive antibodies in SLE, the innate immune system is increasingly appreciated as playing an important role in the pathogenesis of SLE (35). Activating Fcγ receptors are highly expressed on monocyte-derived dendritic cells (mo-DC) and macrophages. Immune complexes formed by DNA or RNA/HMGB1 and IgG can activate these innate immune cells through their Fcγ receptors to elicit their inflammatory functions (36), which include secretion of type 1 interferon (IFN), TNFα, IL-6 and more. The IFN pathway is a crucial contributor to the disease in some models of SLE. Type I IFN can cause the loss of peripheral tolerance by maturing dendritic cells, which activates T cells that eventually help expand autoreactive B cells (37).

While plasmacytoid DCs (pDCs) make the most type 1 IFN on a per cell basis, monocytes are important IFN producers in SLE because of their abundance compared to pDCs (38). Nucleic acids need to be internalized into monocytes and delivered to TLRs 7 and 9 to trigger the production of IFNs. HMGB1chaperones nucleic acid to endosomal TLRs through a RAGE dependent pathway (39). Porat et al. described two pathways by which SLE serum can activate monocytes, one of which involves HMGB1 delivering its nucleic acid cargo by binding and internalization with RAGE (40). The induction of the IFN signature genes by HMGB1 was shown to be inhibited by a DNA mimetope binding to HMGB1, preventing its interaction with RAGE (40).

PDCs, mentioned above, are specialized to produce high amounts of type I interferons (41). Upon TLR 7 or 9 activation, HMGB1 leaves the nuclei of pDCs and pDCs increase their expression of RAGE as a part of their maturation (42). This creates an autocrine loop which sustains type I IFN production. The pathogenic role of pDCs in SLE is often considered to be a consequence of their production of type I IFNs. Patients with SLE have reduced numbers of pDCs in the blood and an accumulation of pDCs in tissues (43). Reciprocally, IFN regulates HMGB1 secretion by driving its translocation from the nucleus to the cytoplasm prior to release into the extracellular space (44). The activation of the JAK/STAT1 signaling pathway by type 1 IFN stimulation induces this process (45). Additionally, IFN-γ has also been shown to dose-dependently induce HMGB1 release through a TNF-dependent mechanism (46). Taken together, these processes highlight the important role HMGB1 plays in initiating nucleotide-induced IFN signature in SLE.

Neutrophils in SLE can mediate tissue damage and produce IFNs (47). Neutrophils can undergo a specialized form of cell death known as NETosis, releasing neutrophil extracellular traps (NETs), primarily composed of DNA and nuclear proteins. Normally, this process functions to prevent the dissemination of pathogens. In SLE, uncleared NETs can become a source of nuclear self-antigens and immune complexes and complement activation, thereby perpetuating the inflammatory response (48). HMGB1 is both released from neutrophils as a part of NETs and itself can induce the release of NETs. It has been shown that HMGB1 promotes the formation of NETs in mice in a TLR4 dependent manner (49). NETs are confirmed as a source of HMGB1 in SLE patients and are positively correlated with disease progression in lupus nephritis (50).

It is important to note, however, that macrophages, especially those expressing SLE risk alleles, also contribute to SLE (51). Macrophages from SLE patients are defective at clearing apoptotic debris and this delayed clearance can lead to prolonged exposure of autoantigens to the adaptive immune system (52, 53). Monocytes can differentiate into classically activated macrophages (M1) responsible for inflammation and tissue destruction, or alternatively activated macrophages (M2) involved in phagocytosis, inflammation resolution and tissue repair (54). Gene expression profiles have revealed that SLE patients have a biased activation toward M1 macrophages (55). Part of this activation pattern may be explained by the elevated HMGB1 in SLE patients. HMGB1 is known to polarize monocytes into M1-like macrophage phenotypes, skewing macrophage phenotype away from M2-like differentiation and thus decreasing phagocytosis of apoptotic cells (56), leaving patients susceptible to the breakdown of peripheral B cell tolerance and the generation of autoantibodies.

HMGB1 has also been shown to bind to C1q, a component of the classical complement pathway. Leukocyte-associated immunoglobulin-like receptor 1 (LAIR-1) is an inhibitory receptor for C1q on the membrane of many immune cells (57, 58). Son el al showed that HMGB1 and C1q can form a tetramolecular complex on the lipid raft with RAGE and LAIR-1 on monocytes, causing M2-like pro-resolving macrophage polarization (59). In the absence of C1q, or when C1q is low due to immune complex-mediated complement consumption, the high level of HMGB1 in SLE patients may skew to M1 macrophage polarization unchecked, promoting inflammation and further reducing clearance of apoptotic cells, exposing autoantigens and thus creating a favorable environment for the adaptive immune system to generate autoantibodies. It is also reported that in pediatric SLE, elevation of serum HMGB1 and type 1 IFN occur with together decreased expression of LAIR-1 on pDCs, suggesting a potential mechanism for a loss of the inhibitory function of LAIR-1 in SLE (60).

## Role of HMGB1 in Specific SLE Disease Phenotypes

### HGMB1 in Lupus Nephritis

The role of HMGB1 in lupus nephritis (LN) illustrates its central role in linking the innate and adaptive aspects to cause the disease phenotype. Lupus nephritis is an example of immune complex-mediated end organ damage in SLE. It is a frequent complication and an important cause of long-term disability and death in the disease (61). Its etiology, as with that of SLE as a whole, involves the loss of immune tolerance resulting in the production of autoantibodies against nuclear autoantigens, potentially through increased exposure to specific antigens and also through polyclonal B cell activation. These immune complexes activate intrarenal TLRs and IFN signaling, resulting in the local production of proinflammatory cytokines by glomerular endothelium, mesangial cells and macrophages. Damage to renal parenchyma triggers tissue repair mechanisms that lead to glomerulosclerosis and chronic kidney failure (61).

Putterman et al. have shown that in the MRL/lpr mouse model of SLE, anti-DNA antibodies can alter the gene expression in mesangial cells of the kidney, upregulating proinflammatory genes and facilitating kidney damage (62). They further demonstrated that HMGB1 has a synergistic effect with anti-DNA antibodies on this process in a RAGE/TLR2 dependent manner (63). It has also been shown that, through TLR2, HMGB1 can induce proliferation of glomerular mesangial cells. Inhibition of either HMGB1 or TLR2 resulted in the decrease in fibronectin and collagen IV, accompanied by improved glomerular histological changes and sclerosis levels (64).

Renal macrophages from the SLE mice were found to be strong producers of the proinflammatory cytokines TNFα and IL-6, which have been suggested as important pathogenic cytokines in mediating kidney inflammation and damage in SLE (65). HMGB1 overexpression in mice resulted in an increased macrophage proinflammatory cytokine response and increased severity of lupus nephritis, whereas administration of glycyrrhizin, a blocker of HMGB1 had an opposite effect (66). Both *in vivo* and *in vitro* experiments confirmed that HMGB1's enhancement of macrophage response is through receptor RAGE (66). These results demonstrate that HMGB1 has kidney-specific effects in addition to its global contribution to SLE's etiology. Finally, urinary HMGB1 has been shown to differentiate SLE patients with active LN from inactive and from healthy individuals (67), again suggesting high local concentrations of HMGB1 in LN.

### HGMB1 in Neuropsychiatric SLE

Neuropsychiatric systemic lupus erythematosus (NPSLE) is a manifestation of SLE reported in up to 80% of patients. It can affect both the central and the peripheral nervous systems and is mostly characterized by cognitive impairment (68). Damage to the blood-brain-barrier can be seen in NPSLE, which allows anti-DNA antibodies access to the central nervous system (69). A subset of anti-DNA antibodies termed DNRAbs cross reacts with the N-methyl-D-aspartate receptor (NMDAR). In mouse models of SLE, enhanced NMDAR signaling by DNRAbs result in spatial memory impairments (70–73) and in patients, elevated titers of DNRAb correlate with memory impairment. Transient exposure to DNRAb leads to long-term neuronal dysfunction through a 2-stage process with stage 1 involving excitotoxic neuronal death and stage 2 involving microglia activation and neuronal pruning (74). HMGB1 can be secreted by stressed or activated cells, including neurons activated through the NMDAR. Interestingly HMGB1 binds to NMDARs. Nestor et al. showed that dendrites bound to C1q are targeted for destruction, resulting in the deficits in spatial memory seen in SLE. C1q uses HMGB1 as a bridge that connects it to the NMDAR. Both *in vivo* and *in vitro* data showed that NMDAR-HMGB1-C1q complexes formed on dendrites target them for destruction by microglia, which itself is activated by HMGB1 through RAGE/TLR4 (74).

## HMGB1-Based Therapeutics

The standard treatments options for SLE are currently centered around corticosteroids and immunosuppressive drugs with numerous unwanted side effects (75). Therapeutics have shifted toward targeting specific pathways (76). Small molecule inhibitors of HMGB1 such as tashinone IIA derivatives and glycyrrhizin are being investigated with some clinical success (77). The possible therapeutic effects of HMGB1-specific antagonists have also been explored in several preclinical studies. The A box domain of HMGB1 alone can bind to HMGB1 receptors such as TLR2/4 and RAGE without eliciting proinflammatory responses and can, therefore, serve as a potent competitive inhibitor of HMGB1 (18). Administration of HMGB1 A box as an HMGB1 antagonist has been shown to reverse lethality in a model of sepsis (78). The effects of monoclonal HMGB1-neutralizing antibodies have also been investigated in various diseases. In SLE specifically, studies on monoclonal HMGB1 antibodies showed conflicting results, with some experiments demonstrating amelioration of SLE disease phenotypes in MRL/lpr mice and BXSB mice (79, 80) while another finding no effects in disease progression in MRL/lpr mice (81). Contrasting clinical outcomes have led to efforts in inhibiting HMGB1 by other means. In addition to direct inhibition of HMGB1, pathways involving HMGB1 can be harnessed for their anti-inflammatory effects. HMGB1 is known to regulate macrophage polarization through its interaction with RAGE, LAIR-1 and C1q (59). Further studies showed that HMGB1 through a positive feedback loop involving IRF5 increases leukotriene B4 production in activated monocytes while HMGB1 plus C1q increase the production of specialized pro-resolving lipid mediators (82). In the same study, a fusion protein that contains the RAGE-binding fragment of HMGB1 and the LAIR-1-binding fragment of C1q were shown to crosslink the two receptors the same way HMGB1 and C1q do and to exert the same pro-resolving effects both *in vivo* and *in vitro* (82). Recognizing that HMGB1 can be harnessed to enhance tolerogenic properties of the immune system opens up novel opportunities for potential therapeutics.

## Concluding Remarks

HMGB1 has been shown to affect a wide array of disease processes in SLE. Functioning both as a DAMP, HMGB1 is able to exerts its pathogenic effects on both the innate and the adaptive immune systems. HMGB1 also interacts with local cells in the diseased organs in SLE, exacerbating disease progression. Investigating the various effects of HMGB1 on the immune system can be extremely valuable in enhancing our understanding of SLE, and in the development of new therapeutics.

## Author Contributions

TL wrote the manuscript and prepared the figure. MS contributed to the conception and scheme of the manuscript. BD contributed to the conception, supervision, and critical revision of the manuscript.

## Conflict of Interest

The authors declare that the research was conducted in the absence of any commercial or financial relationships that could be construed as a potential conflict of interest.
